# Calibration of an Upconverting Phosphor-Based Quantitative Immunochromatographic Assay for Detecting *Yersinia pestis, Brucella* spp., and *Bacillus anthracis* Spores

**DOI:** 10.3389/fcimb.2020.00147

**Published:** 2020-04-24

**Authors:** Pingping Zhang, Yuanyuan Zhang, Yong Zhao, Yajun Song, Chunyan Niu, Zhiwei Sui, Jing Wang, Ruifu Yang, Dong Wei

**Affiliations:** ^1^State Key Laboratory of Pathogen and Biosecurity, Beijing Institute of Microbiology and Epidemiology, Beijing, China; ^2^Beijing Key Laboratory of POCT for Bioemergency and Clinic, Beijing, China; ^3^Division of Tuberculosis Vaccines, National Institutes for Food and Drug Control, Beijing, China; ^4^Center for Advanced Measurements Science, National Institutes of Metrology, Beijing, China

**Keywords:** immunochromatographic assay, calibration, pathogen, quantitative detection, biodefense

## Abstract

*Yersinia pestis, Brucella* spp., and *Bacillus anthracis* are pathogens that can cause infectious zoonotic diseases with high mortality rates. An upconverting phosphor-based quantitative immunochromatographic (UPT-LF) assay, a point-of-care testing method suitable for resource-limited areas, was calibrated to quantitatively detect pathogenic bacteria. The bacterial purity or activity were ensured via staining methods and growth curves, respectively. Growth assays showed that the classic plate-counting method underestimated bacterial numbers compared with the bacterial counting method recommended by the reference material of the National Institutes for Food and Drug Control, China. The detection results of the UPT-LF assay differed significantly between the bacterial cultures in liquid and solid media and between different strains. Accelerated stability assessments and freeze-thaw experiments showed that the stability of the corresponding antigens played an important role in calibrating the UPT-LF assay. In this study, a new calibration system was developed for quantitative immunochromatography for detecting pathogenic bacteria. The results demonstrated the necessity of calibration for standardizing point-of-care testing methods.

## Introduction

Despite having lower sensitivities than sequencing and PCR methods, immunochromatographic assays (ICAs) are extensively employed for point-of-care testing in many developing countries and offer the first-line of defense against acute serious pandemic zoonotic diseases with high mortality rates. Such diseases include the plague, brucellosis, and anthrax, whose etiologic agents are *Yersinia pestis, Brucella* spp., and *Bacillus anthracis*, respectively. These pathogens have caused countless deaths during historical pandemics and are distributed worldwide in natural foci, each having natural reservoirs (such as rodents and fleas for *Y. pestis*). In addition, these pathogenic bacteria are easily acquired and reproduced for use as bioterrorism agents and pose a great potential threat to public health. Timely diagnosis and therapy is crucial for saving patients' lives owing to limited therapeutic strategies and poor prognoses. Etiological diagnoses are especially critical because of the remarkable resemblance to clinical syndromes of diseases caused by bioterrorism agents and other factors. ICAs are rapid and portable and are widely used to detect pathogenic bacteria on-site for surveillance in natural foci and handling of public health emergencies, impeding the transmission of these diseases to the greatest possible extent.

Correctly estimating the accuracy, detection limits, and comparability of ICAs is difficult because analyzing qualitative results based on naked-eye observations via the present colloidal gold ICA is unsuitable for refined calibration research. Upconverting phosphor-based technology based on the lateral flow assay (UPT-LF), a newly developed ICA method for quantitative detection, enables the establishment of a reliable calibration method for ICAs. UPT-LF assays for the detection of pathogenic bacteria have been developed and evaluated in our laboratory, and include assays to detect *Y. pestis* (Yan et al., 2006), *B. anthracis* spores (Li et al., 2006), *Brucella* spp. (Qu et al., 2009), *Vibrio cholerae* (Hao et al., 2017), *Burkholderia pseudomallei* (Hua et al., 2015a; Liang et al., 2017), *Francisella tularensis* (Hua et al., 2015b), *Escherichia coli* O157:H7 (Wang et al., 2007), *Vibrio parahaemolyticus*, and seven *Salmonella* spp. (Zhao et al., 2016). Our UPT-LF assays showed robust performance using many clinical and environmental samples such as biochemical reagents and various types of powders and viscera samples (Zhang P. et al., 2014; Hua et al., 2015a,b), as well as field water samples (Hao et al., 2017). After the samples are loaded onto the strip, upconverting phosphor (UCP) particles that have first been combined with monoclonal antibodies (mAbs) against bacteria can capture the corresponding bacteria and are then captured by the other antibodies against bacteria fixed onto the test band (T band), while the remaining UCP-mAbs complexes are captured by goat anti-mouse IgG on the control band (C band). The visible light emitted by the UCP particles under the excitation of infrared light can be transformed into electric current signals. The ratios of the T- and C-band signals, or the T/C ratio, are used as the detection results in practical applications and are proportional to the bacterial concentration (Yang, 2019). Considering the principle of UPT-LF for quantitative bacterial detection, calibration of the assay should rely on the standard curve, using the detection value and target bacterial concentrations as parameters.

However, the plate-counting method, the current international gold standard for counting bacteria, only counts live cells (Breed and Dotterrer, 1916) and thus may be unsuitable for evaluating the actual abilities of the ICA because antigens remain on the surfaces of dead cells, thus making calibration of the ICA difficult (Zhang X. et al., 2014; Gorsuch et al., 2019). Counting via microscopy or optical density measures are more exact methods for all cells. According to the Beer–Lambert law, absorbance measured via spectrophotometry can be used to quantify substances (Klumpp et al., 2009; Meyers et al., 2018), while the bacterial concentrations are proportional to the optical density at 600 nm (Dalgaard et al., 1994; Stevenson et al., 2016). The National Institutes for Food and Drug Control (NIFDC, China) developed a spectrophotometric method to determine the concentrations of *Y. pestis* (Wei et al., 2009), *Brucella* spp. (Li et al., 2011), and *B. anthracis* (Wei et al., 2010) according to the bacteria enumerated directly via direct microscopic counts. Reference Materials for the Bacterial Content of Plague Vaccines, Brucellosis Vaccines and Anthrax Vaccines (Approval No. National Biological Reference [2013] 0049, issued by the China Food and Drug Administration), were further developed, while the optical densities for three tubes containing glass fibers correspond to three concentrations for the three bacteria, and the standard curves of the two parameters can be used to quantify the bacteria in samples (Table 1). In addition, the culture methods, including the various media and culture conditions (such as fluid medium or a solid plate) may significantly influence bacterial growth because the cellular components may differ under different conditions such as growth rate (Klumpp et al., 2009; Scott et al., 2010; Klumpp and Hwa, 2014) or temperature (Labrie et al., 2005), while heterogeneous populations may result from mutations (Schaechter and View From Here Group, 2001). In this study, a UPT-LF assay for detecting *Y. pestis, Brucella* spp., and *B. anthracis* was calibrated using the NIFDC bacterial reference material, and the influences of culture conditions and various strains were studied to form a solid foundation for calibrating and standardizing immunochromatographic assays.

**Table 1:** Congruent relationship between the optical density at 600 nm of the NIFDC reference material and bacterial concentrations.

**Reference Material (RM)**	**Corresponding concentration of *Y. pestis* (× 10^**8**^ cell mL^**−1**^)**	**Corresponding concentration of *Brucella* spp. (× 10^**8**^ cell mL^**−1**^)**	**Corresponding concentration of *B. anthracis* (× 10^**8**^ cell mL^**−1**^)**
RM with low optical density	4.3	18	0.4
RM with Medium optical density	6.2	25	0.55
RM with high optical density	8.4	34	0.72

## Materials and Methods

### Materials

The strains used to calibrate the detection were *Y. pestis* 91001, *Brucella abortus* S19, *B. anthracis* Sterne, and other strains of *Y. pestis, B. anthracis, Bacillus atrophaeus, Bacillus subtilis*, and *Bacillus cereus*. All strains were preserved in our laboratory. The culture media, lysogeny broth (LB, including tryptone and yeast extract), Hiss agar, and brain heart infusion (BHI) were purchased from Oxoid, Ltd., Beijing Land Bridge Technology Co., Ltd. (Beijing, China), and Becton Dickinson and Company (New Jersey, USA), respectively. Gram stain kits were purchased from Beijing Solarbio Sciences & Technology Co., Ltd. (Beijing, China). Reference Materials for the Bacterial Content of Plague Vaccine, Brucellosis Vaccine, and Anthrax Vaccine were obtained from the NIFDC, China. The SpectraMax M2 Microplate Reader from Molecular Devices Co., Ltd. was used to measure optical density.

**Figure 1:**
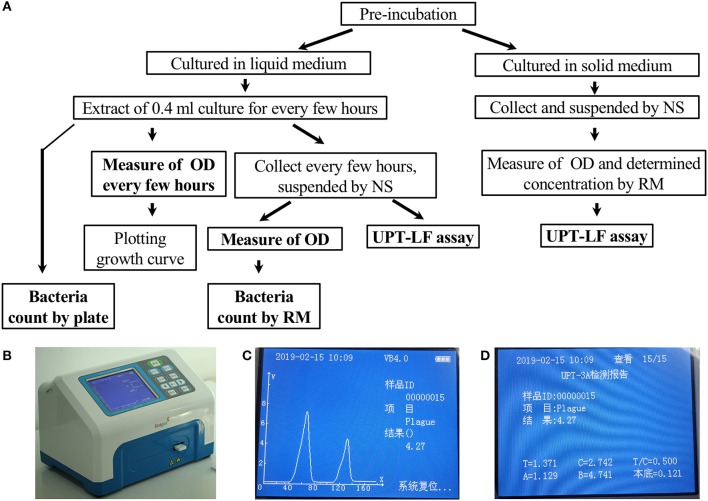
The criteria program and an illustration of the UPT-LF assay. **(A)** Program for the UPT-LF assay criteria. **(B)** The photograph of UPT biosensor scanning of a strip. **(C)** The detection result shows the concentration of bacteria. The signal peak on the left is derived from the control band, and that on the right is derived from the test band. **(D)** Information stored by the UPT-LF biosensor including time, sample ID, detection target, concentration of the target, the value of the signal for the test band and control band, T/C ratio, as well as the parameters for standard curves (y = Ax + B, the logarithm of T/C-cutoff as x and the logarithm of concentration as y) for quantitation of the concentration of target bacteria, including the cut-off, A and B. Some information, with the exception of the detection result, was recorded in radiofrequency identification devices (RFID) for each commercial strip, and this can be revised through a new record after calibration.

UPT-LF strips for detecting *Y. pestis, B. abortus*, and *B. anthracis* were developed by our laboratory, and the UPT-LF detection kits, including strips with the corresponding sample-treatment buffer, were produced in workshops with air cleanliness up to the one million level of good manufacturing practices at Beijing Hotgen Biotechnology Co., Ltd. Radiofrequency identification devices (RFID) in the UPT-LF strips recorded the strip parameters, including the detection target, cutoff, and information from the standard curves (A and B value), which were automatically recognized by a UPT-3A biosensor. The UPT-3A biosensor reads the signals and exports the detection results of the UPT-LF assay (Figures 1B–D). The monoclonal antibodies for *Y. pestis* on the UPT-LF strip were prepared by injecting purified F1 antigen into BALB/c mice. The monoclonal antibodies for *B. abortus* and the polyclonal antibodies for *B. anthracis* on the strip were selected based on injecting whole inactivated bacterial cells into mice or goats; therefore, the information on their corresponding antigens was unclear. The standard curves of the UPT-LF strips in the commercial detection kit for quantifying *Y. pestis, Brucella* spp., and *B. anthracis* were based on the detection results for *Y. pestis* 91001, *B. abortus* S19, and *B. anthracis* Sterne cultured in liquid medium, as well as the numbers determined via the classic plate-counting method.

### Criteria for Culturing and Identifying the Bacteria

The program used to determine the UPT-LF assay criteria is summarized in Figure 1A.

*B. anthracis, B. atrophaeus, B. subtilis*, and *B. cereus* spores were prepared and identified. For preincubation, 0.2 mL of bacteria were incubated in 5 mL of LB broth at −80°C, then cultured at 37°C with shaking at 200 rpm for 15.5 h. The bacteria were collected via centrifugation at 8,000 rpm for 5 min, then inoculated onto nutrient agar plates from Roche bottles (~30 × 15 cm) after being suspended in 2 mL of LB broth. The bacteria were cultured for 7–8 days, then suspended in normal saline to prepare the spores. To determine the spore quality, the bacteria were scraped into 0.2 mL of normal saline and stained with malachite green and crystal violet-iodine on slides treated with 3% hydrochloric acid. For the malachite green staining, a mixture of 0.1 mL of bacteria and 0.1 mL of malachite green were boiled at 100°C for 25 min, then 75% ethyl alcohol was used as a wash buffer after heat-fixing and staining. Using a Gram stain kit, the bacteria were stained with crystal violet for 1 min, washed, then soaked in iodine solution for 1 min, and washed in 20 mL of water.

*Y. pestis* 91001 and *B. abortus* S19 were used for plotting growth curves. For preincubation, 0.2 mL of bacterial solution containing 30% glycerol preserved at −80°C was inoculated into tubes containing 5 mL of medium, then cultured at 37°C with shaking at 200 rpm for different times. The optical densities at 600 nm (OD_600_) were detected three times each, using LB medium as the blank for *Y. pestis* 91001 and BHI medium as the blank for *B. abortus* S19. The preincubation time that resulted in an optical density of ~1.0 was considered the optimal preincubation time for the culture. For incubation, 1 mL of preincubated culture was inoculated into three Erlenmeyer flasks containing 19 mL of LB medium, and the OD_600_ of the culture for all three flasks was measured via spectrophotometry for 1 or 2 h.

The optical density of the bacteria was measured both in normal saline and culture medium. Three parallel Erlenmeyer flasks containing *Y. pestis* 91001 were preincubated for 16 h (to an OD_600_ of ~1.0) and then cultured. At 2, 4, 6, 8, and 12 h, 0.4 mL of the bacterial culture was extracted and divided into two samples. One sample of the bacterial culture was used to directly measure the optical density with LB as a blank, and the other sample was centrifuged at 8,000 rpm at 4°C for 8 min to harvest the bacteria. The supernatant was then removed and the pellet was suspended in saline solution to the same volume as that used to measure the optical density. The optical density was then measured using saline solution as a blank. Similarly, three flasks of *B. abortus* S19 were preincubated for 17.5 h and cultured for 7.3, 8.3, and 9.5 h in BHI medium or normal saline, then the optical densities of the bacteria from the three flasks were measured using BHI medium and normal saline as the blanks, respectively.

### Comparing Counting Methods for *Y. pestis* 91001 and *B. abortus* S19

The standard curves for counting *Y. pestis, Brucella* spp., and *B. anthracis* were plotted using the OD_600_ for each NIFDC reference material for different bacterial contents (including low, medium, and high concentrations) and measured three times using a spectrophotometer. The optical densities were determined for *Y. pestis* 91001 after a 16 h preincubation and 6 h incubation, *B. abortus* S19 after a 17.5 h preincubation and 7.3 h incubation, and *B. anthracis* stern spores after being collected and suspended in normal saline. After measuring their optical densities, their concentrations were calculated from the standard curve of the NIFDC reference material.

For the plate-counting method, the bacterial cultures mentioned above for plotting the standard curves of NIFDC reference material were serially diluted 10-fold according to pre-estimated concentrations based on experience. The dilutions containing the final three low concentrations were inoculated onto Hiss agar plates containing 5% goat blood for *Y. pestis*, BHI plates containing 5% goat blood for *B. abortus* S19, and LB plates containing 5% goat blood for *B. anthracis* stern spores. The concentrations of each of the three flasks were measured for repetition. Plates containing 30–300 colony-forming units were used to calculate the bacterial concentrations in the original flask cultures based on the fold dilutions.

### UPT-LF Assay for Detecting *Y. pestis* 91001 and *B. abortus* S19 Cultured in Liquid and Solid Media

First, different solid media were used to evaluate the bacterial growth states, then 0.2 mL of *Y. pestis* 91001 after a 16 h preincubation was smeared and cultured on LB and Hiss agar plates, while *B. abortus* S19 after a 17.5 h preincubation was smeared on BHI, BHI containing 5% goat blood, LB, and LB containing 5% goat blood. All bacteria were then cultured at 37°C. After 24 h, the bacteria on the plates were scraped into tubes containing 0.8 mL of normal saline. The bacteria in the liquid medium were then prepared according to the above-mentioned method for plotting the standard curves of NIFDC reference material.

After harvesting from liquid or solid media and suspending in normal saline, the optical densities of the bacteria were measured, then their concentrations were determined from the standard curves of the NIFDC reference material. After dilution with sample-treatment buffer, 0.1 mL of *Y. pestis* 91001 at 1 × 10^5^, 1 × 10^6^, 1 × 10^7^, and 1 × 10^8^ cells ml^−1^, *B. abortus* S19 at 1 × 10^6^, 1 × 10^7^, 1 × 10^8^, and 1 × 10^9^ cells mL^−1^, and *B. anthracis* Sterne at 1 × 10^5^, 1 × 10^6^, and 1 × 10^7^ cells mL^−1^ were directly applied to the UPT-LF strips. To read the signals on the strips, the detection results, T/C ratios, and bacterial concentrations were retrieved directly using the UPT-3A biosensor.

### Inclusivity of the UPT-LF Assay

After plate culturing and calibration of the concentrations using the NIFDC reference material, the *Y. pestis* strains 91001, EV, 0614F, Otten, Tjiusidej(R), MII, and M23 at concentrations of 1 × 10^4^, 1 × 10^5^, 1 × 10^6^, 1 × 10^7^, and 1 × 10^8^ cells mL^−1^, *Brucella suis* S2, *Brucella melitensis* M5, and *B. abortus* S19 and 104M at 1 × 10^6^, 1 × 10^7^, and 1 × 10^8^ cells mL^−1^, and *B. anthracis* Sterne, A16R, and CTN-1 at 1 × 10^5^, 1 × 10^6^, and 1 × 10^7^ cells mL^−1^ were diluted with sample-treatment buffer and detected via the UPT-LF assay. All *Y. pestis* and *Brucella* spp. strains were also detected via a colloidal gold immunochromatographic assay (CG-ICA). For *Y. pestis* detection, 1, 10, and 100 ng mL^−1^ of F1 antigen were extracted and purified from the bacteria for use as a positive control and were tested via the UPT-LF assay.

### Antigen Stability

*Y. pestis* and *Brucella* spp. (1 × 10^8^ cells mL^−1^) were prepared and divided into three samples: the first was used as a control, the second was stored at 37°C for 5 d, and the third was freeze-thawed three times. After treatment, 1 × 10^5^, 1 × 10^6^, and 1 × 10^7^ cells mL^−1^ of *Y. pestis* 91001, EV, 0614F, Otten, and Tjiusidej(R), and 1 × 10^7^ and 1 × 10^8^ cells mL^−1^ of *B. abortus* 104M, *B. suis* S2, and *B. melitensis* M5, were diluted with sample-treatment buffer and detected via the UPT-LF assay.

### Statistical Analysis

All experiments were repeated three times. The statistical data were compared via paired-sample *t*-tests with a 95% confidence interval using Origin 8.0 software from OriginLab Corporation (MA, USA).

## Results

### Criteria for Culturing and Identifying Bacteria

**Figure 2:**
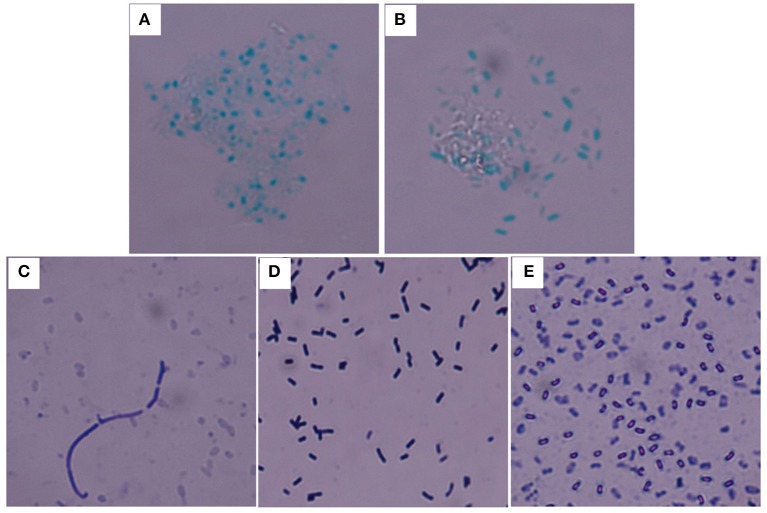
Staining of the spores and vegetative forms of *B. anthracis*. *B. anthracis* Sterne **(A)** and *B. cereus* 41 **(B)** spores stained with malachite green. **(C)** Vegetative form of *B. anthracis* Sterne among spores stained with crystal violet-iodine. **(D)** Vegetative form of *B. subtilis* stained with crystal violet-iodine. **(E)** *B. subtilis* spores stained with crystal violet-iodine.

*Y. pestis* 91001, *B. abortus* S19, and *B. anthracis* Sterne were used for the calibration. The bacterial purity and activity, including the cellular integrity and surface constituents, were critical for evaluating the accuracy of the immunoassay. This study primarily focused on the bacterial quality for calibration because the specificity of the UPT-LF assay for detecting *Y. pestis, B. abortus* S19, and *B. anthracis* had been demonstrated in a previous study (Zhang P. et al., 2014). For *B. anthracis, B. atrophaeus, B. subtilis*, and *B. cereus*, the bacterial spores can be dyed green by malachite green stain, whereas for the Gram-positive bacteria, their vegetative forms were recognized via crystal violet-iodine using a Gram stain. Both staining methods were used for identification to ensure the purity of spores after preparation (Figure 2). The spore spheres were viewed by microscopy (Figures 2A,B). The spore culturing time should be extended if vegetative forms of the bacteria are visible, which appear as long rods after Gram staining (Figure 2C). Both the vegetative form and spores of *B. subtilis* could be recognized after culturing and Gram staining (Figures 2D,E). The *B. anthracis* spores with the highest purity were used as the foundation for calibrating the UPT-LF assay.

**Figure 3:**
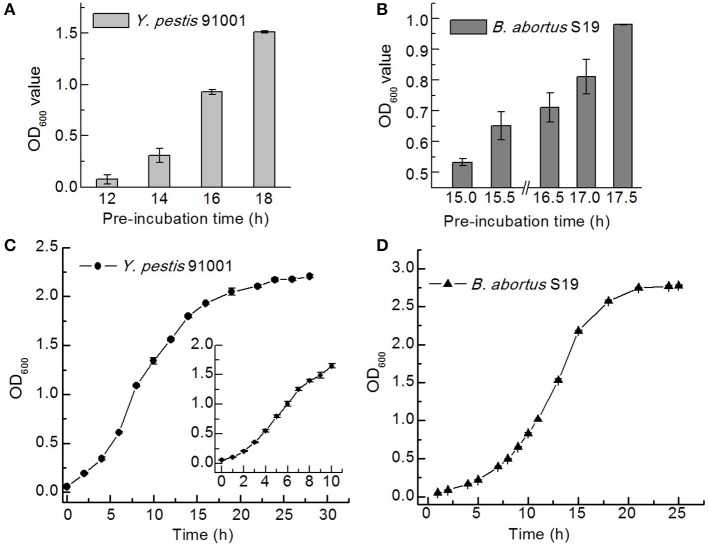
Preincubation times and growth curves for *Y. pestis* 91001 **(A,C)** and *B. abortus* S19 **(B,D)** in the LB and BHI media.

After determining the optimal preincubation times by measuring the optical density at 600 nm, growth curves were plotted for *Y. pestis* 91001 in LB medium and *B. abortus* S19 in BHI medium at 37°C to ensure the bacteria were in an active state. The optimal preincubation times for *Y. pestis* 91001 and *B. abortus* S19 were 16 and 17.5 h, respectively, and the logarithmic phases lasted 3–10 and 7–15 h, respectively (Figure 3).

### Comparison of the Optical Densities in Normal Saline and Culture Media

**Figure 4:**
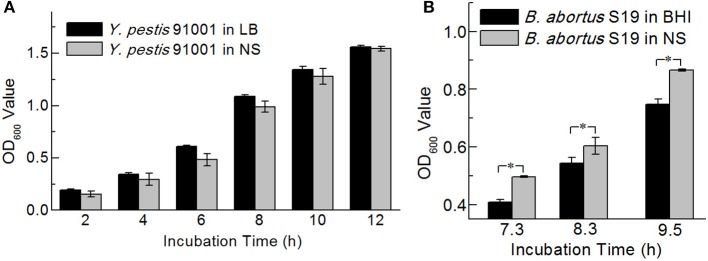
Optical densities of *Y. pestis* 91001**(A)** and *B. abortus* S19 **(B)** in LB or BHI medium and normal saline. Asterisks denote significant differences in OD value (*t*-test; *p* < 0.05; *n* = 3).

The optical densities of the bacteria in normal saline were measured because normal saline is the blank control used for the NIFDC reference material. After 16 and 17.5 h of preincubation, the optical densities of the *Y. pestis* 91001 and *B. abortus* S19 cultures incubated for different times were determined, using LB and BHI as blanks. The same cultures were also resuspended in normal saline after centrifugation and the optical densities were measured using normal saline as a blank. Paired-sample *t*-tests at the 0.05 level showed minimal differences between the two groups in terms of optical density for *Y. pestis* 91001, whereas the optical densities differed significantly for *B. abortus* S19 (Figure 4). Interestingly, the optical density of *Y. pestis* 91001 in medium was higher than that in normal saline and vice versa for *B. abortus* S19, emphasizing that the optical densities differ for different bacteria and media.

### Comparison of Bacterial Counting Methods

**Figure 5:**
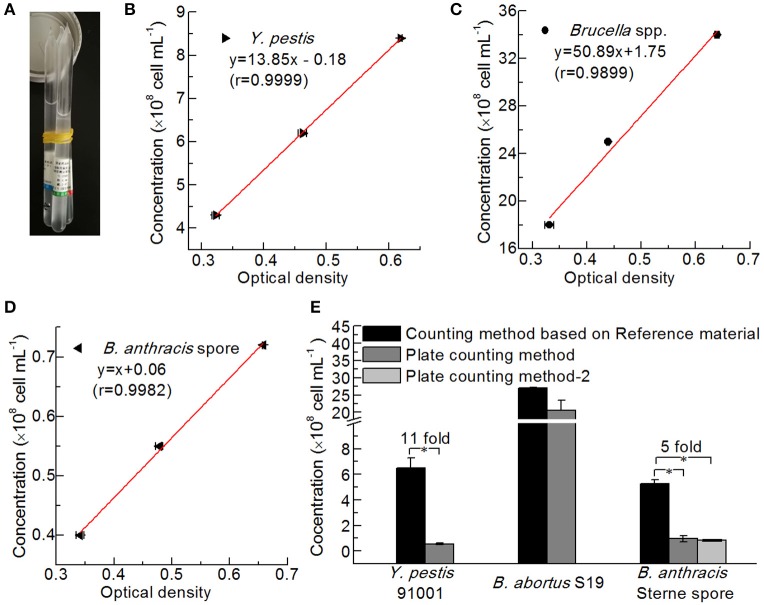
Comparison of different bacterial counting methods. **(A)** Reference Material for Bacterial Content of the Plague Vaccine, Brucellosis Vaccine, and Anthrax Vaccine from NIFDC. Standard curves of the reference material for counting *Y. pestis* **(B)**, *Brucella* spp. **(C)**, and *B. anthracis* **(D)** were plotted based on optical density. **(E)** Comparison of the bacterial concentrations determined by the reference material method and the plate-counting method (Hiss agar plate containing 5% goat blood for *Y. pestis* 91001, BHI plate containing 5% goat blood for *B. abortus* S19, and LB plate and LB plate containing 5% goat blood for *B. anthracis* Stern spores). Asterisks denote significant differences in bacterial concentrations determined by the counting methods (*t*-test; *p* < 0.05; *n* = 3).

The bacteria were counted via two methods, namely, plate counts and counting based on the NIFDC reference material (Figure 5A). Standard curves were constructed showing the optical densities at 600 nm for each reference material and the concentrations of *Y. pestis, Brucella* spp., and *B. anthracis* (Figures 5B–D), and the concentrations of the bacterial solutions, were confirmed when their optical densities were measured. The concentrations determined by the two counting methods were compared (Figure 5E). Although the method using the reference material yielded slightly higher numbers than the plate-counting method for *B. abortus*, the differences between the two methods for *B. abortus* S19, and between the two plate types for *B. anthracis* Sterne were not significant. Paired-sample *t*-tests revealed that *Y. pestis* 91001 and *B. anthracis* Sterne spores differed significantly in number, and the numbers obtained with the reference material method were 11- and 5-fold higher than those of the plate-count method. The differences between the two methods for counting *Y. pestis* may have partly resulted from the culture temperature being 37°C for the production of the F1 antigen, whereas 28°C is the best temperature for preparing the reference material. *B. anthracis* Sterne spores are considered more stable than those of other bacteria; however, we suspended and stored the spores in normal saline before counting, which resulted in low concentrations being determined via the plate-counting method because the NaCl in normal saline significantly influenced the number of viable cells (Wei et al., 2012). Thus, the number of dead cells in a culture may significantly influence the detection results of a quantitative immunoassay.

### Differences in the Detection Results of the UPT-LF Assay After Culturing in Liquid or Solid Media

**Figure 6:**
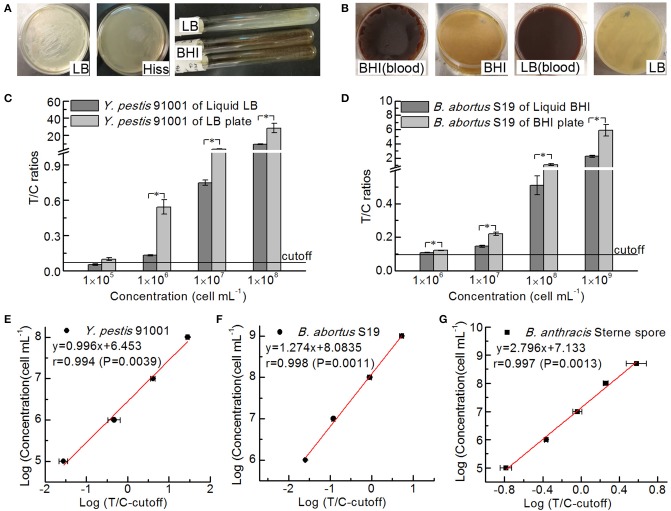
Differences in the UPT-LF detection results for *Y. pestis* 91001, *B. abortus* S19, and *B. anthracis* Sterne cultured in liquid and solid media. The growth conditions for *Y. pestis* **(A)** and *B. abortus* **(B)** differed on different solid media. UPT-LF detection results for *Y. pestis* differed significantly between liquid LB broth and LB plates **(C)** and for *B. abortus* S19 between the liquid BHI medium and BHI plates **(D)**. All bacterial concentrations were determined using the NIFDC reference material, and the standard curves for the UPT-LF assay for quantifying *Y. pestis* 91001 **(E)**, *B. abortus* S19 **(F)**, and *B. anthracis* Sterne spores **(G)** were plotted after calibration. Asterisks denote significant differences in UPT-LF results for bacterial culture of liquid and solid medium (*t*-test; *p* < 0.05; *n* = 3).

First, *Y. pestis* 91001 and *B. abortus* S19 were cultured on different types of solid media. The growth rates of *Y. pestis* 91001 on LB plates were higher than those on LB inclined-plane medium, BHI inclined-plane medium, and Hiss agar plates (Figure 6A), while the growth rates of *B. abortus* S19 on BHI containing blood were higher than those on BHI and both LB plates (Figure 6B). However, bacteria cultured on plates containing goat blood were not used for the UPT-LF assay because goat blood cells may affect the results of the assay. *Y. pestis* 91001 and *B. abortus* S19 cultured on LB and BHI plates, as well as in the corresponding liquid media, were used for the UPT-LF assays after their concentrations had been determined from the NIFDC reference material (Figures 6C,D). Most of the T/C ratios for *Y. pestis* 91001 and *B. abortus* S19 grown in the liquid medium were significantly lower than those grown on solid medium, demonstrating that the amounts of certain antigens on the bacteria differed under different culture conditions. This phenomenon was consistent with previous reports (Klumpp et al., 2009; Klumpp and Hwa, 2014), inferring that culture methods should be screened for immunoassay calibration. After calibration, the standard curves for quantifying *Y. pestis* 91001, *B. abortus* S19, and *B. anthracis* Sterne spores (Figures 6E–G) were plotted to calculate the concentrations. The quantification results showed approximately 1.5- and 0.5-fold differences between the results with or without calibration for most *Y. pestis* 91001 and *B. abortus* S19 concentrations (data not shown).

### Inclusivity Evaluation for the UPT-LF Assay

**Figure 7:**
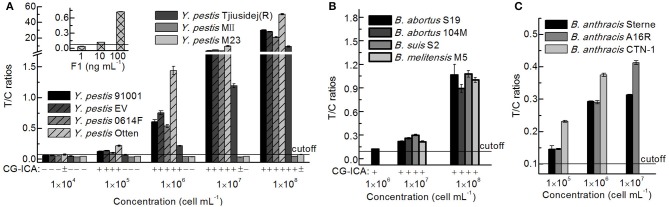
Detection results for different *Y. pestis, Brucella* spp., and *B. anthracis* strains. **(A)** Detection results for *Y. pestis* stains and the F1 antigen via a UPT-LF assay and CG-ICA. **(B)** Detection results for *Brucella* spp. strains via UPT-LF and CG-ICA methods. **(C)** Detection results for *B. anthracis* strains via a UPT-LF assay.

After culturing on plates, the concentrations of bacteria were determined according to the reference material, and seven *Y. pestis*, four *Brucella* spp., and three *B. anthracis* strains of different concentrations were detected via the UPT-LF assay (Figure 7). The sensitivities of the assay for *Y. pestis* strains 91001, EV, Otten, 0614F, and Tjiusidej(R) were 10^5^ cells mL^−1^, while MII and M23 yielded negative results. *Y. pestis* M23 is a F1-negative strain that lacks the virulence determinant of the F1 envelope antigen (Burrows and Bacon, 1958; Seguin et al., 1987). Although its F1 genes (*caf1, caf1A, caf1M*, and *caf1R*) are 100% identical to those of *Y. pestis* 91001, *Y. pestis* MII is an attenuated strain with fewer surface proteins than those of other *Y. pestis* vaccine strains (Lei et al., 1997). *B. suis* S2, *B. abortus* 104M, and *B. melitensis* M5 can be detected with similar T/C ratios to that of *B. abortus* S19, which was the same for *B. anthracis* A16R and CTN-1 compared with *B. anthracis* Sterne. For most stains, the qualitative results were consistent between the CG-ICA assay and the UPT-LF assay (Figure 7). Considering the inclusivity of the immunoassay, the quantitative performance of the bacterial concentrations using immunoassays should be defined more precisely for calibration. Using standard curves for each bacterial strain is impractical for an on-site detection method. The standard curves of the UPT-LF assay for quantifying *Y. pestis* were determined for *Y. pestis* 91001 in our laboratory. In conclusion, for calibration, the standard curves for quantifying concentrations were based on certain bacteria, while the true concentration for a specific bacterium hinges on the amount of F1 antigen on its surface.

### Antigen Stability on the Bacterial Surface Corresponded With the Results of the UPT-LF Assay

**Figure 8:**
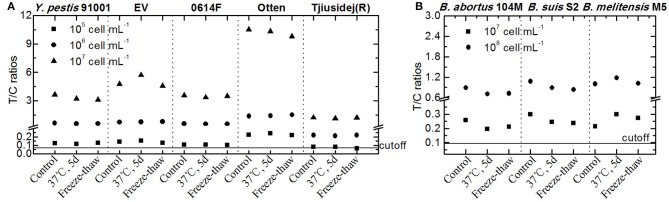
Detection results for the UPT-LF assay for *Y. pestis* **(A)** and *Brucella* spp. strains **(B)** in accelerated stability assessments.

The stability of the antigen to which an immunoassay is targeted is a pivotal factor that influences the accuracy of the detection results. Because degradant levels are more than 100-fold greater under higher temperatures than under lower temperatures according to the Arrhenius equation (Waterman, 2011), we evaluated the stability of antigens stored at 37°C for 5 days, as well as after three freeze-thaw cycles to assess the accelerated stability (Figure 8). The recovery rates, or the proportionality between the T/C ratios of the bacteria after treatment and those of the control, were all higher than 97% (Table 2). In conclusion, the treatment had little influence on the results of the UPT-LF assay for *Y. pestis* strains 91001, EV, 0614F, Otten, and Tjiusidej(R), or *Brucella* spp., at various concentrations.

**Table 2:** Recovery rate^*^ for each strain under various parameters in accelerated stability assessments.

**Bacteria**	**Parameter**					
	**37°C, 5 d**			**Freeze-thaw**		
	**10^**5**^ cell mL^**−1**^**	**10^**6**^ cell mL^**−1**^**	**10^**7**^ cell mL^**−1**^**	**10^**5**^ cell mL^**−1**^**	**10^**6**^ cell mL^**−1**^**	**10^**7**^ cell mL^**−1**^**
*Y. pestis* 91001	98.8%	98.9%	99.2%	100.7%	99.2%	99.0%
*Y. pestis* EV	101.4%	100.2%	101.1%	98.8%	100.5%	99.8%
*Y. pestis* 0614F	99.5%	99.5%	99.6%	98.3%	99.6%	99.8%
*Y. pestis* Otten	100.8%	100.2%	99.9%	99.8%	100.6%	99.6%
*Y. pestis* Tjiusidej(R)	100.9%	99.5%	99.4%	-	99.9%	99.8%
	**10**^**7**^ **cell mL**^**−1**^	**10**^**8**^ **cell mL**^**−1**^		**10**^**7**^ **cell mL**^**−1**^	**10**^**8**^ **cell mL**^**−1**^	
*B. abortus* 104M	96.2%	98.2%		97.4%	98.3%	
*B. suis* S2	97.7%	98.5%		97.3%	98.1%	
*B. melitensis* M5	104.2%	101.2%		103.1%	100.1%	

* *Recovery rate = (T/C ratio under various parameters)/(T/C ratio of the control)*.

## Discussion

In this study, we developed a comprehensive immunochromatographic calibration method for quantitatively detecting bacteria. Our findings confirmed that: (1) bacterial purity and activity could be guaranteed via staining and growth curves, respectively. (2) Bacterial numbers should be confirmed using the NIFDC reference material, not plate-counting methods. (3) Antigen amounts differed significantly between cultures in liquid and solid media; therefore, the culture method should be fixed for calibration. (4) The amounts of specific antigen differed between each *Y. pestis, Brucella* spp., and *B. anthracis* strain, therefore the stains should be specified for calibration. (5) The stability of the corresponding antigens indicated the stability of the immunoassay, with surface antigens greatly influencing the assay results. After calibration, the UPT-LF assay yielded more exact bacterial quantification results, and all of the factors that influenced the detection results in this calibration method should be considered when performing other immunochromatographic assays.

Because pathogenic bacteria responsible for serious infectious diseases can induce infections in several cell types and lead to death or disease breakouts, qualitative detection results are often sufficient. However, calibration of a quantification immunoassay may still have practical significance for diagnosing bacteria. First, it can help standardize pathogen detection and precisely evaluate the detection limits and inclusivity of an immunoassay. Second, the bacterial concentrations should be exactly determined for vaccine production, which can be achieved by a rapid quantification immunochromatographic method. Third, such an assay could be applied to detect foodborne bacteria, whose detection limits differ among various foods.

In this study, the sensitivities of the UPT-LF assay for *Y. pestis, Brucella* spp., and *B. anthracis* were about 1 × 10^4^-10^5^ cells mL^−1^, 1 × 10^4^-1 × 10^5^ cells mL^−1^, 1 × 10^3^-1 × 10^4^ cells mL^−1^, respectively, which were at least 10-fold lower than that of the UPT-LF assay developed in our laboratory (Li et al., 2006; Yan et al., 2006; Qu et al., 2009). This may be because commercial, mass-produced UPT-LF strips were used for the calibration method for practicality reasons, but these strips may be of inferior quality, thereby resulting in decreased sensitivities. Nevertheless, UPT-LF detection in which the sample is directly added to the strip without any need for pre-treatment is especially useful as a field assay in resource-limited areas of developing countries, and meaningful calibration results can still be obtained. For example, the UPT-LF assay is practical for the surveillance of infected animals in natural foci for *Y. pestis, Brucella* spp., and *B. anthracis*, when the samples being tested (such as dead animals) contain a large number of bacteria. Furthermore, the sample volume for the UPT-LF assay is 100 μL, in other words, only 10^3^ bacterial cells are applied to the strip when the concentration of the bacterial sample is 10^4^ cells mL^−1^. For comparison, the sensitivities of real-time or isothermal recombinase PCR are about 10 copies per reaction for *Y. pestis* (Qu et al., 2010), *B. anthracis* (Antwerpen et al., 2008; Bentahir et al., 2018), and *Brucella* spp. (Zeybek et al., 2019), which is equivalent to 2 × 10^3^-1 × 10^4^ cells mL^−1^ in a 1–5 μL sample volume of template for each PCR, thereby enrichment of the target bacteria by culturing or extraction of nucleic acid is often required (Kane et al., 2019). Enrichment of the target bacteria, for example, by centrifugation, could be applied during sample preparation for the UPT-LF assay. Although low concentrations of bacteria could not be tested in this study, all of the factors addressed in the calibration method are relevant to other immunochromatographic assays, including the determination of bacterial purity and activity, the bacterial counting method, culturing conditions, and the different amounts of antigen between strains.

Antibody fabrication limits the inclusivity of immunoassays for bacterial detection. For example, the F1 antigen is one of four virulence determinants for *Y. pestis* determined by the World Health Organization in 1970, but some low-virulence vaccine *Y. pestis* strains, such as M23 and MII, which lack or contain minimal amounts of the F1 antigen, were determined to be negative via the UPT-LF assay fabricated with antibodies against F1. Owing to the great differences in antigens on the surfaces of the various strains for one bacterial type, false-negative results are unavoidable for an immunoassay based on one or a few antibodies. Multiple detection chips based on various monoclonal antibodies against different antigens on the surfaces of some bacteria, as well as new bacterial biomarkers, could be used to promote the development of more accurate immunoassays. The relative limitations of a detection reagent should be illustrated in the instruction manual.

## Conclusion

This study is the first to establish a calibration method for an upconverting phosphor-based quantitative immunochromatographic assay to detect pathogenic bacteria, which included guaranteeing the bacterial purity and activity, accurately counting the bacteria, and determining the antigen amounts under different culture conditions. It also ensured the inclusivity of the assay and the stability of the corresponding antigens. The results indicated that the bacterial preincubation and incubation times, the liquid and solid culturing methods, the bacterial counting method, quantification of different strains of a species, and antigen stability, should all be considered in calibrating immunochromatographic assays. In particular, bacterial strains, culturing and counting methods should be standardized for immunochromatographic assays.

## Data Availability Statement

All datasets generated for this study are included in the article/supplementary material.

## Ethics Statement

BALB/c mice (8 weeks old) and goats were used for the production of antibodies. The Committee of Welfare and Ethics of Laboratory Animals, Beijing Institute of Microbiology and Epidemiology (Beijing, China) reviewed and approved the animal care and experimental protocols concerning the preparation of antibodies (Permit No. IACUC-DWZX-2018- 006). All animal experiments were in compliance with the Guidelines for the Welfare and Ethics of Laboratory Animals of China.

## Author Contributions

RY, DW, and PZ designed the experiments. PZ, YZhang, YZhao, YS, CN, ZS, and JW performed the experiments. PZ, RY, and DW analyzed the data and wrote the manuscript.

## Conflict of Interest

The authors declare that the research was conducted in the absence of any commercial or financial relationships that could be construed as a potential conflict of interest.
